# Making a Difference—Positive Effect of Unilateral VIM Gamma Knife Thalamotomy in the Therapy of Tremor in Fragile X-Associated Tremor/Ataxia Syndrome (FXTAS)

**DOI:** 10.3389/fneur.2018.00512

**Published:** 2018-06-27

**Authors:** Piotr Alster, Dariusz M. Koziorowski, Mirosław Za̧bek, Sebastian Dzierzȩcki, Jacek Ma̧dry, Karolina Duszyńska-Wa̧s, Hanna Grygarowicz, Justyna Zielonko, Leszek Królicki, Andrzej Friedman

**Affiliations:** ^1^Department of Neurology, Medical University of Warsaw, Warsaw, Poland; ^2^Department of Neurosurgery, Medical Centre for Postgraduate Education, Warsaw, Poland; ^3^Nuclear Medicine, Medical University of Warsaw, Warsaw, Poland

**Keywords:** FXTAS, tremor, gamma knife (GK), radiosurgery, thalamotomy

## Abstract

Fragile X Tremor Ataxia Syndrome (FXTAS) is a syndrome based on expansion of the repeats of CGG triplets. The symptoms include action tremor and cerebellar gait ataxia. Additionally symptomatology of FXTAS may be associated to parkinsonism, executive function deficits, dementia, neuropathy and dysautonomia. We present a case of a patient who after 20 year history of progressive tremor and ataxia, was diagnosed after genetic examination as mutation of FXTAS. For the treatment of tremor the patient underwent Gamma Knife (GK) thalamotomy. Reduced tremor on the right side and improvement in everyday activities were observed in the outcome of the treatment. GK thalamotomy, in the context of this patient, did not significantly affect the ataxia.

## Introduction

Fragile X-Associated Tremor Ataxia Syndrome (FXTAS) is an adult-onset neurodegenerative disorder which is often associated with action tremor and cerebellar gait ataxia (1). Clinical features of FXTAS include parkinsonism, executive function deficits, dementia, neuropathy, and dysautonomia. These symptoms, especially when combined, are completely disabling and can cause severe detriment to the quality of life of patients. A T2-weighted magnetic resonance imaging (MRI) of affected individuals usually reveals the Middle Cerebellar Peduncle (MCP) sign, which is a marked symmetric bilateral hyperintensity within the middle cerebellar peduncle. Other areas observed as hyperintense include the splenium of the corpus callosum, pons, insula, and periventricular white matter. Pharmacotherapies are currently unsuccessful in making a substantial improvement in the quality of lives of those suffering with FXTAS.

## Background

Non-pharmacological interventions for the treatment of FXTAS include: Deep Brain Stimulation (DBS), Gamma Knife Radiosurgery (GK), Focal Ultrasound (FUS) and Radiofrequency (2–13) with DBS and Radiofrequency the most popular amongst current literature (2–6, 13). With respect to positive outcomes of these treatments, bilateral stimulation with DBS of the ventro-oralis posterior (VOP) thalamic nucleus and zona incerta (ZI) have been documented (14).

Bilateral stimulation of the ventral intermediate thalamus (VIM) has shown to alleviate tremor and ataxia in FXTAS in a long-term analysis (2). Further, although unsustained, similar outcomes were observed intraoperatively with an additional DBS lead into the ventralis oralis anterior (VOA) nucleus and the VOP region (2–6). Insertion of a second VIM DBS lead has shown to worsen gait (2–6), whereas stimulating the posterior subthalamic area has shown the contrary (4). A study on a Polish family affected by FXTAS, confirmed by molecular analysis, highlighted the possible outcome of the deterioration of cognitive abilities after DBS surgery (7). Needless to say, the risks of side effects due to DBS for the treatment of FXTAS cannot be eliminated. However, it does seem that DBS presents an optimistic approach in the treatment of FXTAS (2–6).

GK radiosurgery has a documented efficacy in the treatment of tremor (8–10), and Higuchi et al. specifically propose disabling tremors as an indication for GK in their recent article (8). Unilateral GK treatment seems to be regarded as most effective in the literature (8–10), whereas for patients with bilateral tremor GK is regarded as unbeneficial. The efficacy of GK in the treatment of intractable tremor is estimated at 70–90% (8). The maximum dose of radiation in GK is estimated as 120–180 Gy. Bilateral thalamotomy remains insufficiently researched and lacks publication in contemporary literature (8). One publication has focused on GK treatment for a patient with refractory Parkinson's disease (PD) tremor, with tremor assessment measured by the Fahn-Tolosa-Marin Rating Scale (FTRS) (9). Results showed that 70.0% of examined patients experienced full tremor relief, while an optimistic 93.9% had a significant reduction of tremor (9). Although GK has great potential in the treatment of symptoms associated with various syndromes, there remains the standing issue of a 3-6 month delay of tumor suppression.

MRI-guided focused ultrasound ablation (MRI-FUS), although only approved for the treatment of tremors associated with refractory essential tremor (ET), has been analyzed in the context of several other indications such as tremor in PD, dystonia, epilepsy, brain tumor and neuropathic pain (11, 12). The low risk of side effects after repeated treatments using this method, as well as possible reversible effect on neural function, are noted advantages of MRI-FUS (12). As an FDA-approved method for the treatment of ET, this method of lesioning presents a significant angle in the therapy of various diseases including FXTAS cases.

## Case presentation

**Figure 1:**
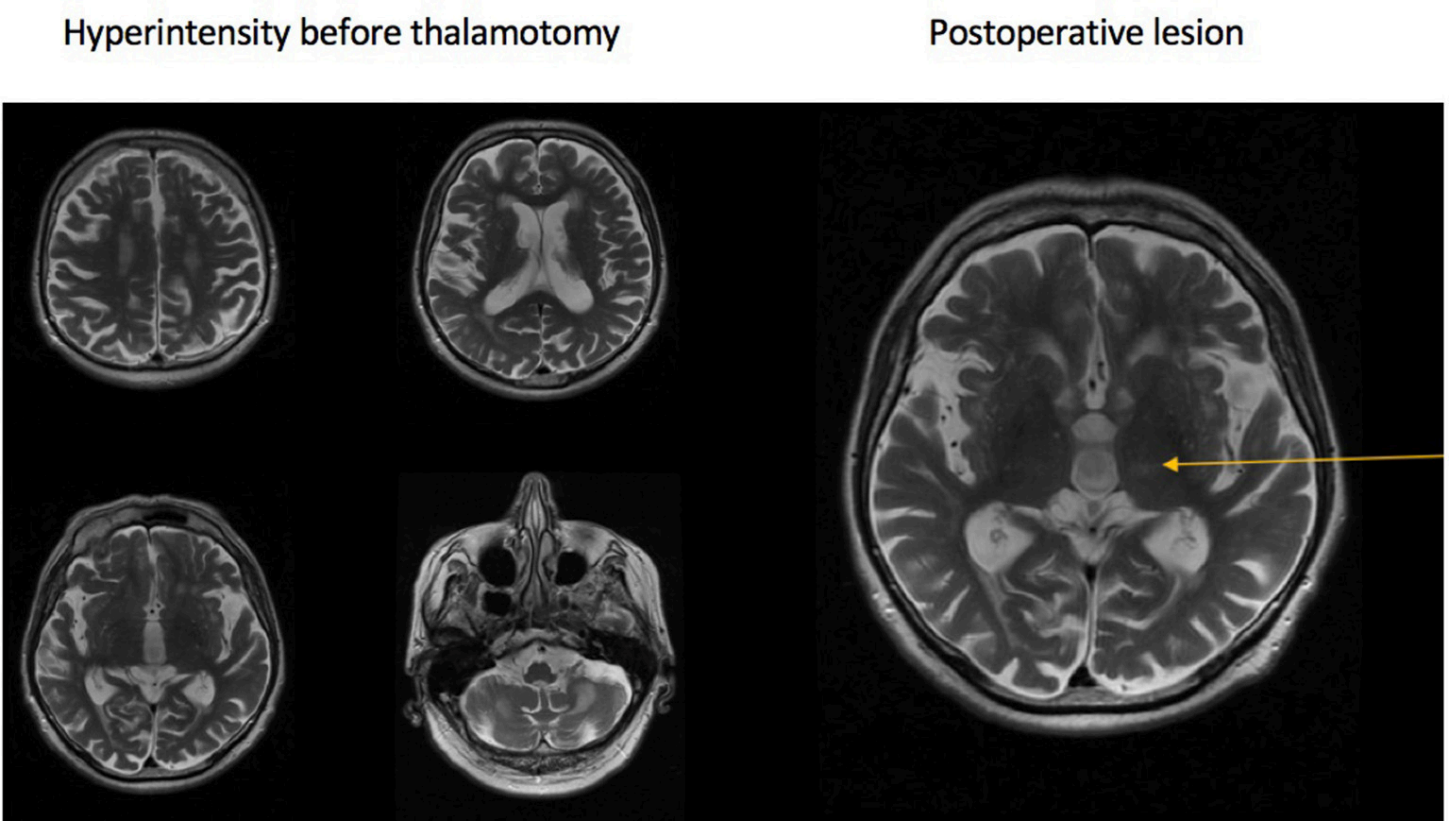
Hyperintensities in the brain and cerebellum **(left)**, postprocedural lesion **(right)**.

A 73-year-old male patient was admitted to the Department of Neurology due to tremors of the head, neck, and upper limbs, and moderate tremor of lower limbs. Initial symptoms appeared 20 years prior, primarily as a kinetic tremor of the left upper and lower limbs. During a neurological examination, the patient additionally presented dysarthria, paresis of the right facial nerve, brisk reflexes on the right side, bilateral dysmetria, dysdiadochokinesis, truncal ataxia, ataxia of lower limbs which was more prominent on the left side, and unstable gait. Psychological examination exposed mild cognitive impairment and deficits of executive functions. An MRI in 2016 revealed bilateral hyperintensity in the MCP and white matter of cerebellar hemispheres (Figure 1).

The combination of intention tremor, ataxia, the MCP sign, and gait instability of patient's son and daughter instigated diagnosis of FXTAS. Use of the Amplide X FMR1 PCR kit (Asuragen) for extended molecular examination revealed permutations in the *FMR1* gene (between 60 and 85 ± 5 repeats of CGG), and confirmed the diagnosis of FXTAS with expansion of CGG repeats in the *FMR1* gene.

Due to insufficient effects of pharmacological treatments (primidone and propranolol) to treat the tremors, radiosurgical thalamotomy using GK on the VIM of the left thalamus was performed. The Leksell Model G stereotactic coordinate frame (Elekta AB, Sweden) was attached to the patient's head. High-resolution MRI were acquired using a 1.5T scanner, and contrast-enhanced T1 and T2 images taken at 1 mm. The anterior commissure (AC), the posterior commissure (PC), and the third ventricle were identified (Figure 1).

Stereotactic radiosurgical thalamotomy was performed using the Leksell Gamma Knife Perfexion model (Elekta AB, Sweden). The target was localized to the VIM nucleus of the left thalamus. Final isocenter coordinates were selected by means of GammaPlan Software (Elekta AB, Sweden), and were set in the following manner: (x coordinate) 16 mm lateral to the AC-PC line which constituted 50% of the width of the III ventricle plus 11 mm; (y coordinate) 8 mm anterior to the PC; and (z coordinate) 3 mm superior to the AC-PC line.

Final optimization of the x coordinate was safely adjusted in order not to exceed the 18 Gy dose in the posterior limb of the internal capsule. A maximum dose of 130 Gy was delivered with a single, fully opened 4-mm collimator. Control examination was done 6 months after GK thalamotomy. Improvement was observed 4 months after the procedure.

Comparison of tremor intensity before and after the procedure was done using the FTRS and the Tremor Rating Scale (TRS) (Table 1). The case presented in this study showed improvement in both rating scales (FTRS 54 – before and 44 – after, TRS – 28 – before and 14,5 after). The improvement was observed in the right side corresponding to the lesion (Videos 1, 2). This represents the most significant change in the context of the head tremor, postural tremor of the upper extremity and daily activities. Neurological examination showed that features such as dysarthria, central paresis of right facial nerve, bilateral dysmetria, dysdiadochokinesis, lower limb ataxia remained unchanged or insignificantly changed.

**Table 1:** Fahn-Tolosa-Marin Tremor Rating Scale (FTRS) motor score and Tremor Rating Scale (TRS) changes.

**FTRS scale**	**Right side**		**Left side**		**TRS scale**	**Before GK January 2017**	**After GK September 2017**
	**Before GK January 2017**	**After GK September 2017**	**Before GK January 2017**	**After GK September 2017**			
Upper extremity tremor	AR: 0 POST:1 ACT/INT: 1	AR: 1 POST: 0 ACT/INT: 1	AR: 1 POST: 2 ACT/INT: 1	AR: 1 POST: 2 ACT/INT: 3	Head tremor	2	1
					Face tremor excluding mandibular	1	0
Lower extremity tremor	AR: 0 POST: 0 ACT/INT: 1	AR: 0 POST: 0 ACT/INT: 1	AR: 0 POST: 1 ACT/INT: 0	AR: 0 POST: 1 ACT/INT: 0	Tongue tremor	0	0
					Voice tremor	1	0
Drawing (0 – normal, 4 – unable to complete drawing (results A+B+C)	6	4	9	12	Tremor in standing position	2	1
					Spiral drawing	3	2
Pouring (0 – normal – 4 unable to pour water)	2	1	4	4	Writing	3	1
					Keeping the pencil	4	1
Writing	4	1		Pouring water	3	2	
Total	15	9	18	23	Total	19	8

*FTRS: 0, none; 1, slight; 2, Moderate amplitude; 3, Marked amplitude; 4, Severe amplitude. TRS – BEFORE AND AFTER: 0, lack of symptoms; 4, most intensive symptoms (AR, at rest; POST, posture, ACT/INT, action/intention)*.

Psychological examination did not present any relevant changes. Physiotherapist's examination revealed certain changes in the period before (2016) and after thalamotomy (2017). In the timed up and go (TUG) test before GK the patient did not present any abnormalities. After GK thalamotomy TUG showed dynamic imbalance. Examination also demonstrated static imbalance after GK thalamotomy, which was not observed before the procedure. Deterioration in certain features of the examination performed by the physiotherapist were associated with the progressive nature of FXTAS. This could also be observed during MRI, in the form of an increased signal in T2-weighted image within both hemispheres of cerebellum and bilaterally within middle cerebellar pedunculi, rather than side effects of GK thalamotomy (13). Approval of the Ethical Committee was not necessary for preparation of this article, as this work is a case study.

## Discussion

According to our best knowledge, this is the first case study of a patient with tremor in FXTAS treated with GK thalamotomy. Certainly, this method of therapy shows certain limitations such as retardation of effect and lack of the possibility of bilateral GK thalamotomy. On one hand, it should be stressed that this method does not enable a neurological assessment during the procedure. However, in this study, GK was used to treat FXTAS with tremors in a case where pharmacotherapy effects were insufficient. Extended analysis of a larger group of patients and a longer observation period could highlight late side effects associated with this treatment. Nevertheless, GK in FXTAS seems to be a promising alternative to DBS in the nonpharmacological treatment of tremor.

## Concluding remarks

A decrease in the intensity of the tremor on the contralateral to the lesion side was observed after GK treatment. The examination was done 6 months after the procedure. Assessments using the FTRS motor score and TRS revealed improvements in the context of tremor on the right side and everyday activities such as pouring, drawing, and writing. There was no significant effect on the ipsilateral side. GK may represent another possibility for safe treatment of tremor in FXTAS cases. The use of this method on a larger group of patients may provide valuable information about the safety and efficacy of FXTAS treatment.

## Ethics statement

Ethical approval not required. This work is a case report. The case of the patient was analyzed retrospectively after the procedure.

## Ethical standards

The mentioned patient gave informed consent prior to the procedure. Written informed consent was obtained from the participant for the publication of this case report and the video in a publicly accessible journal.

## Author contributions

The contribution of each author was as following: PA study design, data collection, data interpretation, acceptance of final manuscript version, literature search. DK and AF study design, data collection, data interpretation, acceptance of final manuscript version. MZ, SD, JM, KD-W, HG, JZ, LK data collection, acceptance of final manuscript version.

### Conflict of interest statement

The authors declare that the research was conducted in the absence of any commercial or financial relationships that could be construed as a potential conflict of interest.
